# Gastrointestinal Mucormycosis in the Setting of Colonic Pseudo-Obstruction

**DOI:** 10.7759/cureus.98414

**Published:** 2025-12-03

**Authors:** Katherine Domenici, Matthew Cappiello

**Affiliations:** 1 Infectious Diseases, Loma Linda University Medical Center, Loma Linda, USA

**Keywords:** colonic pseudo-obstruction, covid-associated mucor mycosis, gastrointestinal mucormycosis, invasive fungal disease, mucor, rhizopus, traumatic diaphragm injury repair, tropical medicine

## Abstract

Mucormycosis is a rare opportunistic infection caused by the fungi order *Mucorales *that can affect multiple different organ systems. A 49-year-old man with a history of poorly controlled diabetes and hypertension presented with acute-on-chronic abdominal pain with profound worsening in the past 24 hours. He was found to have chronic intestinal pseudo-obstruction, with pneumatosis of the transverse colonic wall requiring total abdominal colectomy. Within two weeks after surgery, he developed fundal gastric injury with herniation requiring primary repair with surgical laparotomy. Tissue histopathology post-procedure revealed broad and irregular fungal hyphae, revealed to be *Rhizopus microsporus* on broad-spectrum PCR. Gastrointestinal mucormycosis is an emerging invasive fungal disease, often diagnosed post-mortem due to high mortality rates. Risk factors include noncommunicable chronic diseases such as diabetes and malignancy, as well as communicable diseases, including COVID-19. Surgical debridement is key to improving outcomes, as infection tends to cross tissue planes without adequate source control.

## Introduction

Mucormycosis is a spectrum of invasive fungal diseases caused by the genera of the order *Mucorales* [1]. Currently defined within the phylum *Mucoromycota* and historically defined within the phylum *Zygomycota*, the spectrum of mucormycosis currently includes 261 species and 55 genera, with over 30 species causing disease in humans [2]. Gastrointestinal mucormycosis is one of the most uncommon presentations of the disease, occurring in the context of infectious events, such as abdominal wall soft-tissue infection, or surgical events, such as upper or lower gastrointestinal perforation [3]. Gastrointestinal mucormycosis accounts for approximately 2-8% of all cases [4]. Although the condition is frequently life-threatening in both immunocompetent and immunocompromised patients, diagnosis can occur unexpectedly, often while in the setting of hospitalization for an alternate or secondary reason [5]. Risk factors include chronic comorbidities such as diabetes, renal failure, and primary and secondary immunodeficiencies, as well as acute events such as abdominal trauma or gastrointestinal perforation [3]. In addition, health-associated gastrointestinal mucormycosis has been associated with prolonged intensive care hospitalization [6].

## Case presentation

A 49-year-old man with a history of poorly controlled diabetes and hypertension presented with acute-on-chronic abdominal pain and distention for 24 hours. Review of systems included multiple episodes of nausea and non-bloody nonbilious vomiting, as well as constipation with his last bowel movement four days prior to admission. The patient denied any fevers, chills, or active cough. The patient additionally denied any diarrhoea, hematochezia, or melena. He denied any sick contacts or recent travel, with two domesticated dogs as pets.

On past medical history, the patient was recently diagnosed with an asymptomatic COVID-19 infection one month prior during a hospitalization for ischemic stroke at an outside hospital, with no documentation of him requiring antiviral therapeutics such as remdesivir or corticosteroid therapy. Additionally, he had a working diagnosis of chronic intestinal pseudo-obstruction that was monitored by an outpatient gastroenterologist, with only limited records available. A therapeutic outpatient colonoscopic decompression had been performed one month prior to hospitalization, with only transient improvement. Multiple episodes of loss to follow-up had occurred in the setting of limited health insurance. A gastroenterology follow-up appointment had been scheduled on the day of his arrival at the emergency department. 

On examination, the patient was afebrile and normotensive, with mild tachycardia of 110 beats per minute. Abdominal exam showed distention and tympany, with diffuse tenderness to palpation in all quadrants. The exam was otherwise unremarkable. Labs were unrevealing aside from a mild neutrophilic leukocytosis, normocytic anemia of unclear chronicity, as well as mild to moderate renal insufficiency of unclear duration (Table 1). Although his clinical or biochemical profile did not show evidence of diabetic ketoacidosis with an unremarkable beta-hydroxybutyrate at 0.4mMol/L, he still displayed hyperglycemia up to 400mg/dL, so he was initially managed with a continuous insulin infusion.

**Table 1:** Laboratory investigations on admission. ALP: alkaline phosphatase, ALT: alanine transaminase, AST: aspartate transaminase, eGFR: estimated glomerular filtration rate.

Parameters	Values	Units	Normal range
Sodium	136	mMol/L	136-144
Potassium	5.2	mMol/L	3.6-5.1
Chloride	97	mMol/L	101-111
CO_2_	20	mMol/L	22-32
Urea nitrogen	52	mg/dL	8-20
Creatinine	1.7	mg/dL	.64-1.27
eGFR	49	mL/min	>=60
Glucose	407	mg/dL	74-118
Albumin	3.6	mg/dL	3.5-4.8
Total bilirubin	0.2	mg/dL	0.2-1.2
ALP	158	IU/L	32-91
ALT	24	IU/L	17-63
AST	40	IU/L	15-41
Hemoglobin A1C	12	-	4.6-6.0
White blood cells	11.33	x 10^9^/L	4-10
Hemoglobin	9.5	g/dL	13.5-17.5
Hematocrit	29.6	%	40-53
Platelets	457	x 10^9^/L	150-450

**Figure 1:**
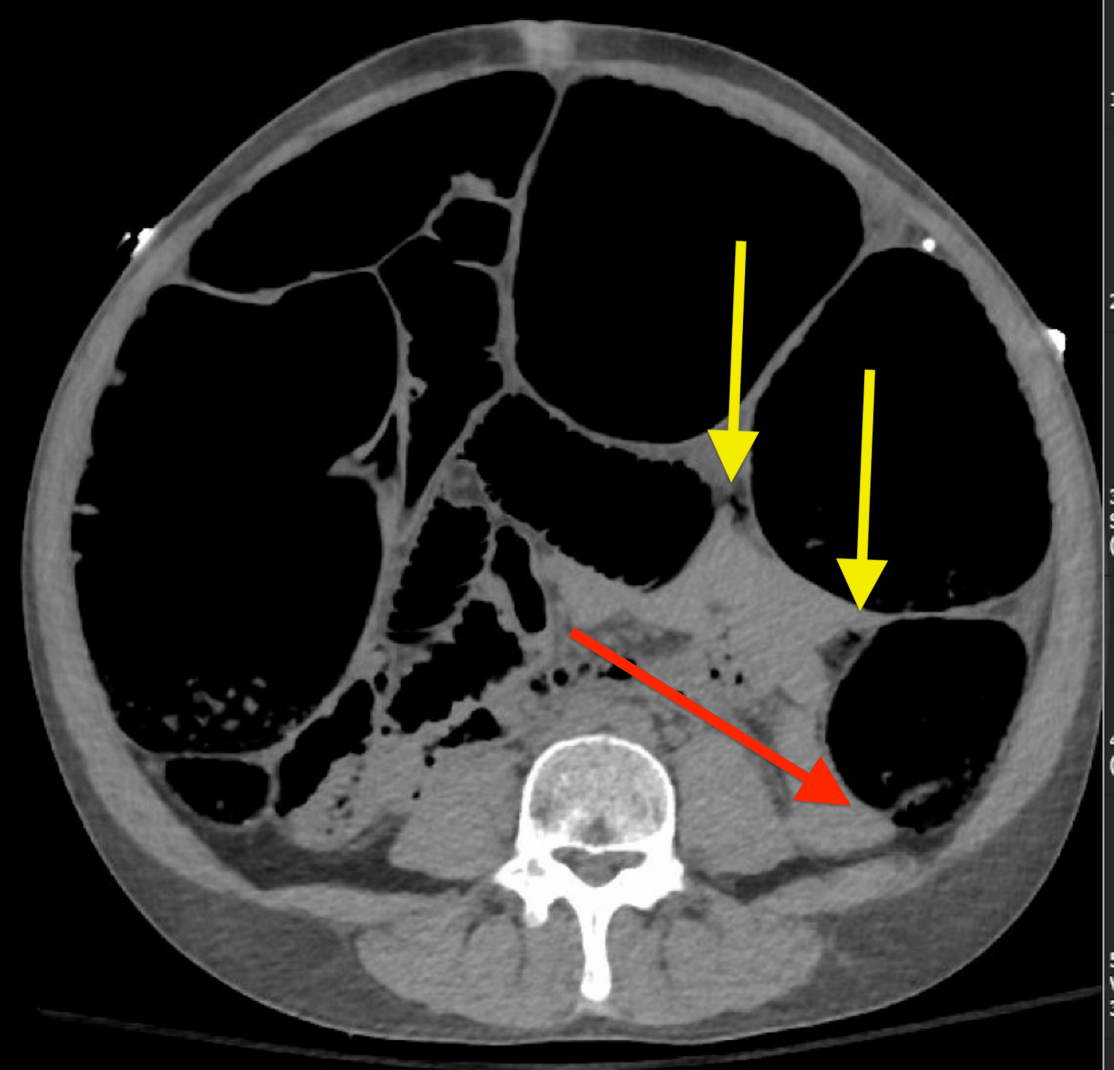
CT abdomen-pelvis on admission, suspicious for pneumatosis coli. Red arrow: Distal tapering of marked colon dilatation near the sigmoid junction, concerning for obstruction, Yellow arrows: multiple foci of air along the descending colon, concerning for visceral perforation.

However, CT angiography of the abdomen and pelvis displayed marked dilatation of the colon, with smooth distal tapering near the sigmoid junction, as well as multiple foci of air suggestive of pneumatosis (Figure 1). 

**Figure 2:**
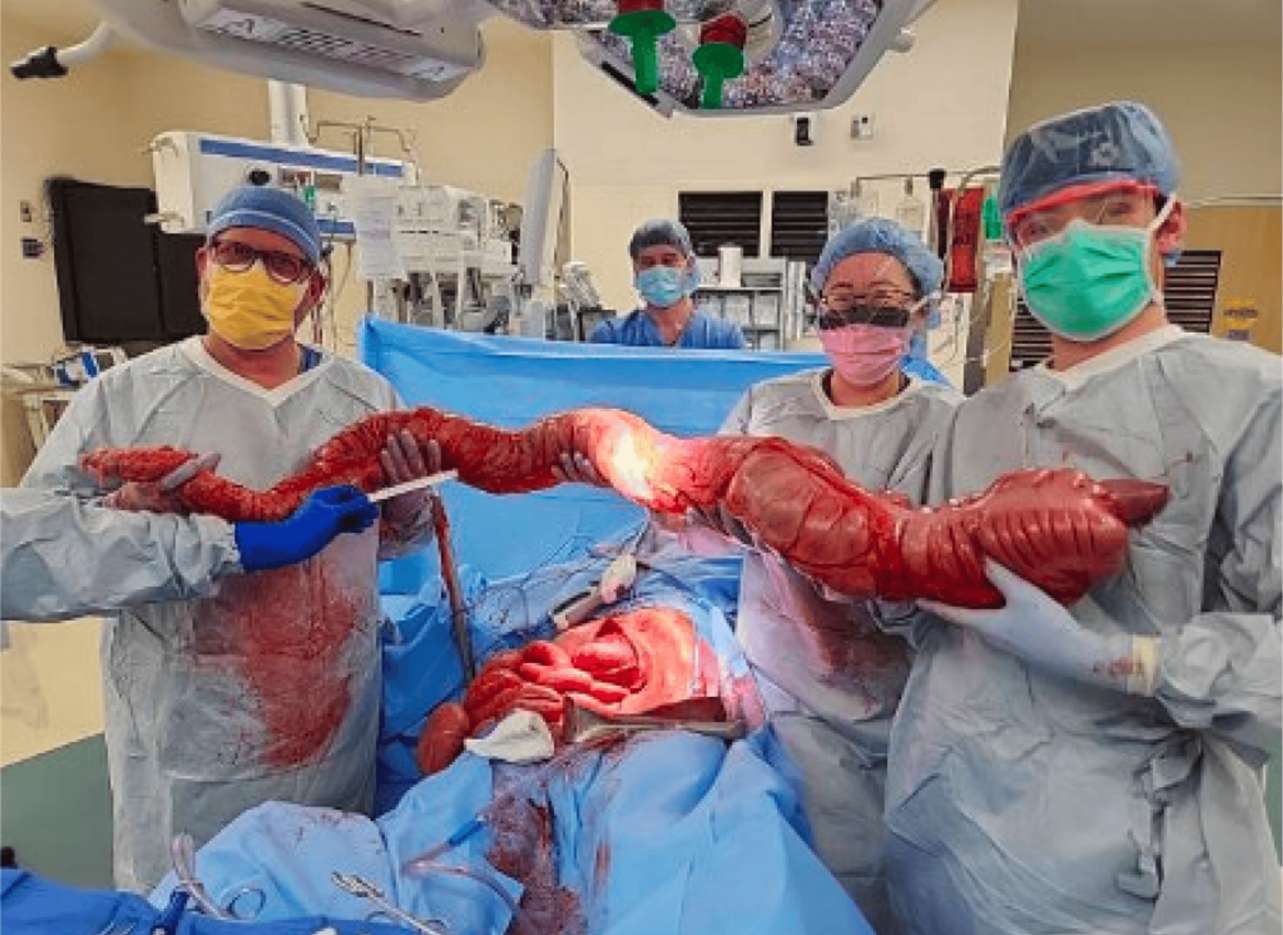
Intraoperative gross pathology of pseudo-obstruction, with enlarged and edematous colon.

The patient was started on piperacillin/tazobactam and red-blanketed to the operating room with the acute care surgery team. Intraoperatively, he was found to have an extensively dilated colon with pneumatosis of the transverse colonic wall (Figure 2). He received a total abdominal colectomy, flexible sigmoidoscopy, and wound vacuum therapy placement. He received a subsequent washout on postoperative day 1, with his fascia closed and a loop ileostomy performed on postoperative day 2. Colonic tissue was sent for pathology, with evidence of mucosal erosion and pneumatosis. Surgical cultures were not performed due to concern for culture result overlap with commensal intestinal flora. 

Concern existed for chronic intestinal pseudo-obstruction, so further workup was performed. He was treated early in his hospital stay with rectal vancomycin and IV metronidazole, but Clostridium difficile antigen and toxin cascade remained negative on two separate tests. HIV fourth-generation antibody and *Trypanosoma cruzi *enzyme immunoassay antibody were negative. He initially remained on the surgical service for intra-abdominal exams and nutritional monitoring.

**Figure 3:**
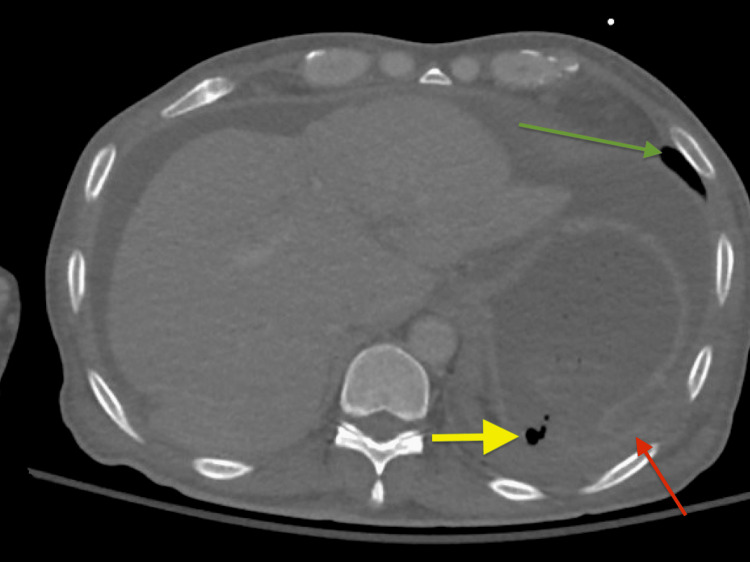
CT chest, showing left diaphragmatic interruption with herniation of gastric fundus into the left hemithorax. Yellow arrow: Foci of air adjacent to the gastric fundus, concerning for gastric rupture, Red arrow: complex left pleural effusion, Green arrow: small-to-moderate volume left-sided pneumothorax.

On postoperative day 15, he developed pronounced epigastric pain with hypotension and increasing leukocytosis. A repeat CT chest showed concern for fundal gastric injury with herniation into the left hemithorax, as well as a loculated left-sided pleural effusion (Figure 3). He received a left thoracotomy and an exploratory laparotomy, with sharp excisional debridement. A defect was noted in the patient’s diaphragm, along with a strangulated and perforated proximal posterior stomach with food contents in the abdomen and left chest cavity. Partial gastrectomy and primary diaphragmatic repair were performed, as well as chest cavity washout and closure with chest tube placement.

Tissue cultures that were obtained during the thoracotomy were positive for *Candida glabrata, Candida albicans, Candida krusei,* *Candida dubliniensis*, and *Lactobacillus gasseri, *which were suspected to be commensal flora. Blood cultures obtained the same day were also positive for *Candida glabrata*. His antimicrobials were then switched to amphotericin B liposome IV and ampicillin/sulbactam to cover broadly for fungal species as well as empyema.

**Figure 4:**
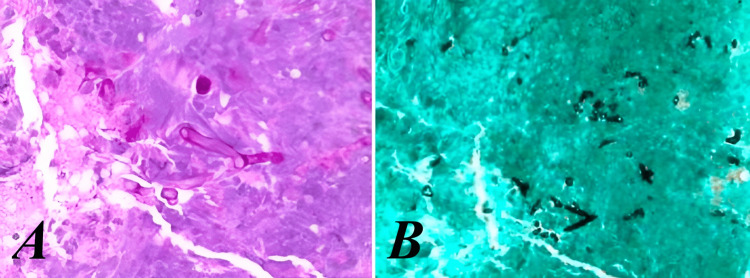
Histopathology of diaphragmatic tissue sample. A. Periodic Acid Schiff stain, showing broad and irregular aseptate hyphae. B. Grocott's methenamine silver stain, showing concern for angioinvasive disease with hyperpigmented fungal elements adjacent to vessel walls.

Pathology findings of the diaphragm were notable for broad, irregular, aseptate hyphae concerning for *Mucorales *(Figure 4)​​​​. Pathology performed retrospective staining on his colonic tissue samples from his initial laparoscopy, which were initially negative for fungal elements. A diaphragm tissue sample was sent to the University of Washington for fungal 16S broad-spectrum PCR, which ultimately detected *Rhizopus microsporus.*

The patient was diagnosed with tissue-invasive gastrointestinal fungal disease, with mucormycosis on specimen identification, in the setting of chronic intestinal pseudo-obstruction as well as medical triggers such as uncontrolled diabetes and antecedent COVID infection. He was started on liposomal amphotericin for the above, with clinical status promptly improving and renal function remaining stable while on therapy. He was eventually transitioned to isavuconazole one and a half weeks after beginning therapy, once the level was confirmed as therapeutic. His chest tube was continued throughout hospitalization and ultimately discontinued prior to discharge, due to concern for concurrent empyema with the above organisms. Ampicillin/sulbactam was continued for a total of seven days from thoracotomy/source control. The isavuconazole was recommended for a minimum of three months, with the possibility of extending therapy if there continued to be signs of infection on repeat imaging. He was discharged with isavuconazole, with plans to follow up with outpatient surgery for eventual ileostomy reversal.

## Discussion

Mucormycosis is a rare opportunistic infection caused by the fungi order *Mucorales *[7]. It can affect any organ system, but the sinuses, oral cavity, and brain are the most commonly affected. Intestinal mucormycosis has a known association with ingestion of *Mucorales* spores. Gastrointestinal mucormycosis is less common, but the number of cases published in PubMed has increased over time [8]. *Mucor* infections generally have a high mortality rate, but vary depending on the site of infection. The mortality of gastrointestinal *Mucor* infections in India is cited between 67-94%. [4] The case described above is unique not only due to the site of infection but also due to the timeline of the patient’s symptoms. Although retrospective staining of this patient's colonic tissue samples was negative for fungal elements, there remained a high suspicion for angioinvasive gastrointestinal mucormycosis. Furthermore, stomach contents were found in the thorax, which suggested that the mucor found in the diaphragm was also infecting the abdominal space.

The inciting cause of the patient's acute-on-chronic abdominal pain was likely multifactorial. His chronic colonic pseudo-obstruction was likely related to his uncontrolled diabetes, with the differential also including neurologic and medication-induced causes. Other common infectious etiologies of pseudo-obstruction were ruled out, including toxic megacolon as well as Chagas intestinal disease [9]. However, there is concern that the patient's interval diagnosis of gastrointestinal mucormycosis was related to visceral gastric perforation with subsequent tissue invasion. Nosocomial acquisition is possible, but less likely in light of his multiple other predisposing conditions to invasive fungal infection. 

Risk factors for this infection include diabetes, chemotherapy, and hematologic malignancies. Diabetes is a risk factor because it affects innate immunity and thus phagocyte function. *Mucor* species harbor ketone reductase, which leads to increased growth in acidic, high-glucose conditions such as diabetic ketoacidosis [10]. Rhino-orbital cerebral mucormycosis is thought to be triggered by GRP78 protein interaction on nasal epithelial cells, which is enhanced by high glucose, iron, and ketone body concentrations [11]. *Mucor *requires free iron, and diabetic ketoacidosis leads to partial dissociation of iron bound to transferrin [12]. Additionally, increased oxygen content is thought to contribute to fungal angioinvasion [10]. It is thought that voriconazole use in the immunosuppressed population predisposes to breakthrough *Mucor* infections, but the exact nature of this association remains unclear [13]. 

Another risk factor is COVID-19 infection, especially in patients with other comorbidities such as uncontrolled diabetes [14]. While our patient did not receive corticosteroid therapy for COVID, a diagnosis of COVID-19 infection itself is associated with hypoxia, hyperglycemia, and a suppressed immune system, which leads to ideal conditions for opportunistic infection [13-14]. Additionally, there is debate in the literature regarding whether home exposures or nosocomial exposures increase the risk of *Mucorales* acquisition in patients afflicted with COVID-19 [15], with differing prevalences of* Mucorales *species depending on the region of incidence in studies from India [13]. The patient described above had multiple of these risk factors, including a recent antecedent COVID-19 infection as well as uncontrolled diabetes.

The high mortality rate associated with gastrointestinal mucormycosis underscores the importance of prompt diagnosis. This is particularly pronounced in the pediatric population, especially neonates suffering from conditions such as necrotizing enterocolitis [16,17]. One clue that a patient has a mucormycosis infection is that their infection crosses tissue planes [18]. The patient in this case had an infection involving the colon and stomach as well as the pleura. Pathologic characterization can help to distinguish colonization from infiltration or invasion [7], and modern techniques such as imprint cytology [19] and PCR [20] can help to improve sensitivity and specificity if clinical suspicion is high. 

Once mucormycosis is diagnosed, treatment involves control of predisposing risk factors, surgical resection of necrotic and infected tissues, and intravenous fungal therapy, including amphotericin. *Mucormycoses* are capable of tissue necrosis and thrombosis, which causes treatment with amphotericin B to remain ineffective without concomitant surgical debridement. Mortality is higher in those who don’t receive surgery [21,22]. Diagnosis must especially not be delayed in resource-limited settings, as the differential for visceral perforation includes both tuberculosis as well as typhoid fever [3]. Additional reduction of predisposing risk factors can include reduction or discontinuation of corticosteroids, as well as control of hyperglycemia. In addition to amphotericin, which is the mainstay of treatment, trials are underway for novel agents such as rezafungin, encochleated amphotericin, and olorofim [23].

## Conclusions

This case highlights several key points that other case reports and articles on intestinal mucormycosis have described. This patient’s infection involved the stomach and pleura through crossing of tissue planes through the diaphragm. Gastrointestinal involvement of mucormycosis has a high morbidity and mortality rate, with surgical debridement essential in improving outcome alongside initiation of amphotericin B. Key populations at risk for gastrointestinal mucor include uncontrolled diabetics as well as patients suffering from COVID-19 infection, both of which are highly prevalent in the general population. 
